# Patient and Physician Satisfaction with Analgesic Treatment: Findings from the Analgesic Treatment for Cancer Pain in Southeast Asia (ACE) Study

**DOI:** 10.1155/2018/2193710

**Published:** 2018-04-18

**Authors:** Dang Huy Quoc Thinh, Wimonrat Sriraj, Marzida Mansor, Kian Hian Tan, Cosphiadi Irawan, Johan Kurnianda, Yen Phi Nguyen, Annielyn Ong-Cornel, Yacine Hadjiat, Hanlim Moon, Francis O. Javier

**Affiliations:** ^1^Department of Radiation Oncology, HCMC Oncology Hospital, Ho Chi Minh City, Vietnam; ^2^Department of Anesthesiology, Faculty of Medicine, Srinagarind Hospital, Khon Kaen University, Khon Kaen, Thailand; ^3^Department of Anesthesiology, Faculty of Medicine, University of Malaya, Kuala Lumpur, Malaysia; ^4^Department of Anaesthesiology, Singapore General Hospital, Singapore; ^5^Department of Internal Medicine, Cipto Mangunkusumo General Hospital (RSCM), University of Indonesia, Jakarta Pusat, Indonesia; ^6^Department of Internal Medicine, Dr. Sardjito General Hospital, Gadjah Mada University, Yogyakarta, Indonesia; ^7^Department of Palliative Care and Pain Management, K Hospital, Vietnam National Cancer Hospital, Hanoi, Vietnam; ^8^Veterans Memorial Medical Centre, Quezon City, Philippines; ^9^APAC LATAM MEA, Mundipharma, Singapore; ^10^Pain Management Center, St. Luke's Medical Center, Quezon City, Philippines

## Abstract

**Aim:**

The aim of this study was to examine patients' and physicians' satisfaction, and concordance of patient-physician satisfaction with patients' pain control status.

**Methods:**

This cross-sectional observational study involved 465 adults prescribed analgesics for cancer-related pain from 22 sites across Indonesia, Malaysia, Philippines, Singapore, Thailand, and Vietnam. Pain intensity, pain control satisfaction, and adequacy of analgesics for pain control were documented using questionnaires.

**Results:**

Most patients (84.4%) had stage III or IV cancer. On a scale of 0 (no pain) to 10 (worse pain), patients' mean worst pain intensity over 24 hours was 4.76 (SD 2.47). More physicians (19.0%) than patients (8.0%) reported dissatisfaction with patient's pain control. Concordance of patient-physician satisfaction was low (weighted kappa 0.36; 95% CI 0.03–0.24). Most physicians (71.2%) found analgesics to be adequate for pain control. Patients' and physicians' satisfaction with pain control and physician-assessed analgesic adequacy were significantly different across countries (*P* < 0.001 for all).

**Conclusions:**

Despite pain-related problems with sleep and quality of life, patients were generally satisfied with their pain control status. Interestingly, physicians were more likely to be dissatisfied with patients' pain control. Enhanced patient-physician communication, physicians' proactivity in managing opioid-induced adverse effects, and accessibility of analgesics have been identified to be crucial for successful cancer pain management. This study was registered at ClinicalTrials.gov (identifier NCT02664987).

## 1. Introduction

Pain associated with cancer is prevalent and negatively affects a patient's psychological and emotional states [1]. Approximately 70–80% of patients with advanced cancer experience moderate to severe pain [2]. The WHO “analgesic ladder” guidelines recommend treating pain in a stepwise approach, starting with nonopioids (step I), then, as necessary, weak opioids (step II), and finally strong opioids (step III) until the patient is free of pain [2, 3].

Patient satisfaction may be used as a key indicator of the effectiveness of cancer pain management in terms of analgesic treatment outcomes [4]. Previous studies have shown that higher patient satisfaction directly influences treatment adherence [5, 6]. Notably, patients' satisfaction levels with pain control vary considerably across different countries and regions. A study conducted across four Northern European countries (Denmark, Germany, Sweden, and United Kingdom) revealed that more than three-quarters (76%) of cancer patients were satisfied with opioid-induced pain relief despite 60% reporting severe pain [7]. In contrast, only 44% of Korean cancer patients with severe pain reported satisfaction with pain control [8].

Despite established cancer pain management guidelines and effective pain medications, a substantial number of patients with cancer pain in Southeast Asia (SEA) still remain inadequately treated for pain symptoms [2, 9]. Although many studies have evaluated patients' satisfaction with pain control status, very few studies have assessed physicians' satisfaction with patients' pain control status in parallel with that of their patients'. As patient-physician relationship also affects patient satisfaction with treatment [5], gaining insights into the alignment of patient-physician satisfaction will hopefully improve future treatment approaches. The objective of the Analgesic treatment for Cancer pain in SouthEast Asia (ACE) study was to provide real-world information on analgesic prescription patterns and patient-reported pain outcomes among cancer patients with pain in SEA. The aim of the current report was to examine patient and physician satisfaction with patient's pain control status in the ACE cohort, and concordance between the two. In addition, we sought to explore the variations in satisfaction with pain control as well as analgesic prescription doses between the 6 participating SEA countries.

## 2. Methods

### 2.1. Study Design and Participants

This was a multicenter, multinational, cross-sectional, observational study conducted between October 2015 and December 2015 at 22 sites in 6 SEA countries (Indonesia, Malaysia, the Philippines, Singapore, Thailand, and Vietnam). Eligible patients were recruited based on these criteria: at least 18 years old; diagnosed with cancer pathologically; outpatients with cancer pain due to cancer itself or its treatment; and treated with any analgesics for more than one month for the management of cancer pain. Patients were excluded from the study if they met any of the following criteria: had an operation for any reason within 3 months; had an oncologic emergency; had any interventional therapy (e.g., nerve block, and neurolytic procedures) related to cancer pain within the past 6 weeks; and current participation in any other interventional clinical trials for cancer treatment or supportive care. All patients provided written informed consent before study enrolment.

Study protocol, case report forms, and documents used for obtaining patients' informed consent were reviewed and approved by the local ethics committee at each study site. All study procedures were conducted in accordance with the Declaration of Helsinki and in compliance with local regulatory requirements.

### 2.2. Study Assessments

Patient demographics, cancer characteristics, treatment histories, and current analgesic prescriptions were obtained from medical records. Questionnaires were administered to patients for self-assessment of worst pain intensity over the past 24 hours (scored on a numeric rating scale (NRS), from 0 (no pain) to 10 (worst pain imaginable) [10, 11]), sleep disturbance due to cancer pain within the past 7 days, quality of life (assessed using the EuroQol Group 5-Dimension Self-Report Questionnaire 3 Level (EQ-5D-3L) system [12, 13]), and patients' satisfaction with pain control status (scored on a 5-point scale: very satisfied, satisfied, acceptable, dissatisfied, and very dissatisfied [14–16]). Attending physicians assessed their satisfaction with their patients' pain control status (scored on a 5-point scale: very satisfied, satisfied, acceptable, dissatisfied, and very dissatisfied) and adequacy of analgesics for pain control (adequate and not adequate).

### 2.3. Statistical Analyses

Of 465 patients recruited into the study, 462 patients met eligibility requirements and were included in the analyses. Patient demographics, cancer characteristics, treatment histories, pain intensities, EQ-5D-3L responses, satisfaction with pain control, and total daily dose of analgesics prescribed were summarized using descriptive statistics. Quantitative variables were summarized as mean (SD) whereas qualitative variables were expressed as number (percentage). Concordance of satisfaction with pain control between patient and physician was evaluated by weighted kappa statistics and McNemar test. *P* values < 0.05 were considered statistically significant. All statistical analyses were performed using R version 3.1.3 (R Development Core Team, Vienna, Austria, 2015).

## 3. Results

### 3.1. Patient Demographics and Characteristics

A total of 465 patients from 6 SEA countries (81 from Indonesia, 100 from Malaysia, 105 from the Philippines, 8 from Singapore, 100 from Thailand, and 71 from Vietnam) were recruited into the study. Three patients did not fulfil eligibility criteria and were excluded from the analysis (two had an operation within three months and one was not treated with analgesics for more than one month), leaving 462 patients in the analysis population.

The analysis population consisted of 46.3% males and 53.7% females, and the mean age of patients was 55.14 (13.39) years. The majority of patients (84.4%) were diagnosed with stage III or IV cancer, and 93.1% had received surgery, radiotherapy, or chemotherapy (Table 1).

More than half of all patients (53.7%, *n*=248) were prescribed a combination of nonopioid and opioid analgesics to manage their cancer pain. On the other hand, 37.0% (*n*=171) received only opioid analgesics, while 9.3% (*n*=43) received only nonopioid analgesics. Of those who received opioid analgesics (*n*=419), more received at least one strong opioid (57.8%) than weak opioids alone (42.2%). Morphine (42.0%, *n*=194) and tramadol (40.9%, *n*=189) were the most frequently prescribed strong and weak opioids, respectively.

### 3.2. Pain Intensity, Quality of Life, and Sleep Disturbance

Based on NRS scoring, mean worst pain intensity over the past 24 hours was 4.76 (2.47). Responses to the EQ-5D-3L questionnaire revealed that 82.3% of patients experienced problems with pain/discomfort, 65.8% with usual activities, 58.2% with mobility, 56.3% with anxiety/depression, and 39.2% with self-care (Table 1). More than half (54.8%) reported sleep disturbance due to pain in the past 7 days.

### 3.3. Patient and Physician Satisfaction with Patients' Pain Control Status

Patient and physician assessment of satisfaction with pain control are presented in Table 2. The majority of patients (60.2%) were either very satisfied (18.6%) or satisfied (41.6%) with their pain control status, while 30.3% found it to be acceptable. A small proportion, however, were dissatisfied (8.0%) or very dissatisfied (1.5%) with pain control. Patient satisfaction with pain control varied significantly across countries (*P* < 0.001); satisfaction was the highest in the Philippines and the lowest in Malaysia (Supplementary Table 1). More physicians (19.0%) than patients (8.0%) reported dissatisfaction with patients' pain control (Table 2). Physician satisfaction with pain control varied significantly across countries (*P* < 0.001); satisfaction was the highest in Singapore and the lowest in Indonesia (Supplementary Table 1).

**Figure 1:**
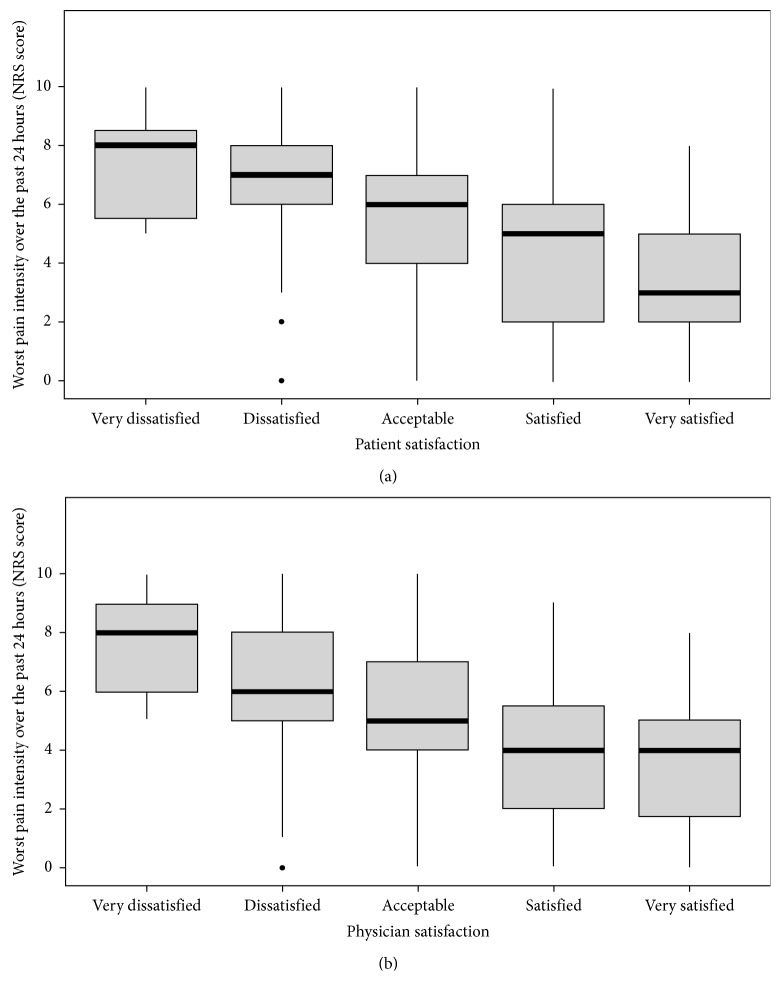
Box plots showing patients' worst pain intensity over the past 24 hours and (a) patients' and (b) physicians' satisfaction with patients' pain control. Horizontal line within the box plot indicates the median; boundaries of the box represent the 25th- and 75th-percentile; filled symbols denote outliers.

Patients who were more satisfied with pain control appeared to have reported lower median pain intensity (very satisfied: median 3, interquartile range 2–5; satisfied: median 5, interquartile range 2–6) than those who were less satisfied with pain control (very dissatisfied: median 8, interquartile range 5.5–8.5; dissatisfied: median 7, interquartile range 6–8; Figure 1(a)). A similar trend was observed for physician satisfaction with patients' pain control (Figure 1(b)).

Overall, 71.2% of physicians described prescribed analgesics to be “adequate” while 28.8% described it to be “not adequate” for pain control. Physician assessment of analgesic adequacy for pain control varied significantly across countries (*P* < 0.001); adequacy was assessed to be the highest in Singapore and the lowest in Indonesia (Supplementary Table 2).

### 3.4. Concordance of Patients' and Physicians' Satisfaction with Patients' Pain Control Status

**Figure 2:**
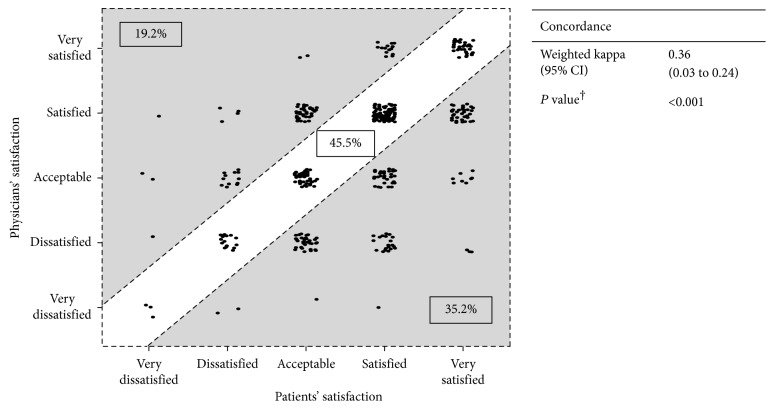
Concordance of patients' and physicians' satisfaction with patients' pain control status (*n*=462). ^†^McNemar's test. CI: confidence interval.

Concordance of patient-physician satisfaction with patients' pain control status is depicted in Figure 2. Satisfaction levels reported by patients and physicians were the same in 45.5% of cases; 19.2% of patients were less satisfied with their pain control compared with their physicians, whereas 35.2% of physicians were less satisfied with their patients' pain control than patients themselves (Figure 2). The disagreement between patients' and physicians' assessment of satisfaction with pain control was significant (*P* < 0.001, McNemar's test).

Evaluating overall concordance of satisfaction with pain control between physicians and patients, a weighted kappa of 0.36 (95% CI, 0.30 to 0.43) was obtained, suggesting low overall agreement on satisfaction with pain control. Concordance of patient-physician satisfaction based on weighted kappa appears to be highest in the Philippines and lowest in Indonesia (Supplementary Table 3).

### 3.5. Doses of Analgesics Prescribed

The median daily doses of prescribed strong and weak opioids, as well as nonopioids, are listed in Table 3. Median daily doses of prescribed opioids, fentanyl, morphine, and tramadol were 0.89 mg, 30.00 mg, and 150.00 mg, respectively. Prescribed median daily doses of opioids were highest in Vietnam and lowest in Indonesia (Supplementary Table 4). On the other hand, median daily doses of prescribed nonopioids, gabapentin, paracetamol, and pregabalin were 900.00 mg, 1300.00 mg, and 150.00 mg, respectively. Prescribed median doses of nonopioids were the highest in Singapore and the lowest in Indonesia (Supplementary Table 5).

## 4. Discussion

Levels of patient and physician satisfaction with analgesic treatment for pain management can be indicators of the effectiveness and appropriateness of current analgesic prescription practices. Using a cohort of SEA cancer patients undergoing analgesic treatment for cancer pain, the present analysis examined levels of patient and physician satisfaction with pain control and analysed the degree of patient-physician agreement with respect to pain control satisfaction. Our key findings were (i) most patients were satisfied with pain control despite unrelieved moderate pain, (ii) low concordance between patient- and physician-reported satisfaction with patients' pain control status, and (iii) physicians tended to be less satisfied with the level of pain control than their patients.

Interestingly, patient satisfaction with pain control was unexpectedly high (60.2%) in our cohort despite clear evidence of unrelieved moderate pain (mean worst pain intensity in the last 24 hours: 4.76 (SD 2.47)). This phenomenon was particularly perplexing given that the majority of patients also reported problems with pain/discomfort (82.3%) and sleep disturbance due to pain (54.8%). Notably, the coexistence of patient satisfaction with pain control and unrelieved moderate to high pain has been reported by others in the literature [16–18]. Some insight into the reasons for this high pain-high satisfaction paradox was provided through patient interviews conducted by Beck et al.; patients conveyed that they expected some pain and believed that their pain cannot be completely relieved or that having unrelieved pain was a choice as they opted not to take their pain medication more frequently [18]. Importantly, cultural perceptions of cancer may also influence the individual's concept of cancer pain; compared to patients of other cultures, Asian patients were inclined to disregard pain [19] and to view their pain as retribution [20] or as an unavoidable consequence of cancer [21]. Others have suggested that the degree of satisfaction with analgesic treatment may be positively influenced by the patients' perception of having control over their pain [22], faster onset of pain relief following administration of pain medication [23], better communication with healthcare professionals [1, 5, 18, 24–26], and prompt management of side effects [18], even though pain continues to be unrelieved. An effective pain management plan coupled with proactive management of analgesic-induced adverse effects would thus be vital for patient satisfaction with analgesic treatment and pain control. Nevertheless, although patient satisfaction with analgesic treatment is a desired outcome, our findings taken together with several others [16–18] suggest that patient-reported satisfaction alone may not be indicative of the patient's pain control status. An all-round assessment of cancer pain and its associated effects (including patient-reported pain intensity, sleep disturbance due to pain, quality of life, and satisfaction with analgesic treatment) may perhaps provide more insight into the pain status of the cancer patient and contribute to better pain management.

A recent study found a discrepancy in reported cancer pain intensity between patients and physicians, suggesting that clinical assessments by patients and physicians may not always be aligned [27]. Indeed, we noted low concordance between patient- and physician-reported satisfaction with pain control in our cohort, with physicians more likely to be dissatisfied than their patients. The specific reasons for physician dissatisfaction with pain control in the present study were not explored. However, high patient loads and the resultant decline in individual patient-contact time may be a plausible source of physician dissatisfaction [28]. Especially in Asia, where shortage of pain management services is perceived to be a barrier to cancer pain management [16], medical consultations may often be too brief for pain specialists to provide quality patient care (e.g., detailed explanation of treatment options and adverse effects and attention to psychosocial aspects of patient complaints). In addition, a large-scale survey of physicians across Asia revealed that physicians generally agreed on the effectiveness of opioids, but excessive regulatory barriers to the use of opioids were identified as a problem by almost half of the physicians surveyed [16]. Based on physician's knowledge of unexplored analgesic options and their potential to effectively manage pain control, we speculate that regulatory barriers may be a contributory factor to physician dissatisfaction with pain control. Indeed, more than 1 in 4 physicians indicated that prescribed analgesics were inadequate for pain control in the present study. As physician satisfaction directly influences quality and delivery of care [29], more needs to be done to establish the sources of physician dissatisfaction with pain management and why they were more likely to be dissatisfied with pain control than their patients.

The small proportion of patients who were less satisfied with their pain control compared to their physicians also contributed to the low patient-physician concordance in satisfaction observed in the present study. Patient-physician contact time is an underrated yet crucial determining factor for both patient satisfaction and physician satisfaction with cancer pain management [28]. Patients who spent less time with their physicians were likely to be less satisfied than their counterparts who had more contact time with the physician [28]. Lack of experience, awareness, and empathy towards cancer pain management on the physician's part could also contribute to perceived poor pain control by patients [30, 31]. Patients may be educated on how to talk about pain with their physicians [32], or on their prescribed therapeutic interventions, such as course of treatment [33], dosage, and concerns about tolerance or addiction [34]. The goal of education would be to raise awareness about self-management of pain and to dispel misconceptions regarding analgesic treatment [35]. Ongoing education is also likely to keep physicians abreast of dynamic cancer pain management approaches [33, 36]. The low concordance between patient- and physician-reported satisfaction with pain control in our cohort highlights the need for improved patient-physician communication about analgesic treatment expectations, as well as pain management education for both patients and physicians in SEA [32, 33].

Amongst the 6 SEA countries included in the present study, Indonesia scored the lowest for physician satisfaction, patient-physician concordance in satisfaction with pain control, and adequacy of analgesics. In addition, median daily doses of the weak opioid, tramadol, and the strong opioids, fentanyl and morphine, were also the lowest in Indonesia amongst all studied countries. Notably, the median daily dose of prescribed morphine was only 20.00 mg in Indonesia—one third of the dose reported to be effective for opioid-tolerant patients with moderate pain [37]. These observations may be the consequence of heavy regulatory restrictions governing the prescription of opioids in Indonesia (e.g., duration of opioid prescription limited to a few days and burdensome procedures for reporting opioid prescription) [38]. Such burdensome regulatory procedures not only limit opioid prescription, but also occupy physician time and results in less time for patient care—a possible contributory factor towards the observed high levels of physician dissatisfaction in Indonesia. These findings highlight the broader picture of availability and/or accessibility of analgesics, particularly those of opioids, which may influence prescription doses and satisfaction levels. National health policies and laws that impose strict regulations on opioid prescription may cause restrictions to opioid availability, inadvertently leading to under-management of pain [39, 40]. A shift towards less regulatory paperwork will allow physicians to focus more on patient care and possibly improve both patient and physician satisfaction with pain management. In less-developed countries whose healthcare infrastructure are under-developed, inaccessibility to opioids is often compounded by high costs and limited variety of opioids [41].

In the present study, study outcomes were primarily evaluated using self-reporting tools. Assessments depending solely on such tools are often one-dimensional and do not capture all aspects of the treatment experience (i.e., pain relief, quality of life, sleep disturbance, and satisfaction with pain control). In addition, owing to the cross-sectional study design, data on all variables were collected only once, and the associations identified between variables were difficult to interpret. The relationship between the variables measured and prescribed analgesics may be better reflected if the questionnaire had been administered before and after analgesic treatment. It is also important to note that variations across countries in patient education levels, types of hospitals (e.g., national, community, or local district hospitals), number of recruited patients (e.g., eight from Singapore compared with 70–100 from other countries), and physicians' education regarding cancer pain management were not taken into account in the comparison of data across countries. More importantly, our findings may not be representative of the whole range of clinical settings for pain management in SEA. Cancer pain management in less-developed regions of SEA is, therefore, likely to be more challenging in reality as issues with healthcare systems and analgesic accessibility and availability still persist.

## 5. Conclusions

The results of our study highlight the complexity of managing cancer pain in SEA. Despite relatively high patient-reported satisfaction with analgesic treatment for pain control, many patients still reported unrelieved pain, problems with quality of life, and sleep disturbance due to pain. In addition, there was low concordance between patients' and physicians' satisfaction with patients' pain control. Successful management of cancer pain will require action on a number of fronts—firstly, noting that patient-reported pain measures are often one-dimensional and do not capture all aspects of the patient's pain control status, an all-round assessment of patients' pain symptoms (including patient satisfaction, pain intensity, sleep disturbance due to pain, quality of life, and adverse effects) would be crucial in assessing the effectiveness of prescribed analgesics. Additionally, one important finding of this study is the evidence of a gap in satisfaction with pain control between patients and their physicians, which suggests a need for further improvements in patient-physician communication about analgesic treatment expectations and pain control. To improve patients' analgesic compliance and quality of life, physicians are also encouraged to proactively assess and manage opioid-induced adverse effects. Finally, national health policies that support and improve the accessibility of analgesics are welcomed so that patients can access the best possible analgesic treatment for their cancer pain.

## Figures and Tables

**Table 1:** Demographics and characteristics of the analysis population (*n*=462).

Age (years)	
Mean (SD)	55.14 (13.39)
Age group, *n* (%)	
18–29 years	17 (3.7)
30–39 years	54 (11.7)
40–49 years	74 (16.0)
50–59 years	139 (30.1)
60–69 years	113 (24.5)
70–79 years	53 (11.5)
80+ years	12 (2.6)
Gender, *n* (%)	
Male	214 (46.3)
Female	248 (53.7)
Cancer stage, *n* (%)	
0	4 (0.9)
I	11 (2.4)
II	32 (6.9)
III	79 (17.1)
IV	311 (67.3)
Not available	25 (5.4)
Metastasis, *n* (%)	
Yes	303 (65.6)
No	144 (31.2)
Unknown	15 (3.2)
Received surgery/radiotherapy/chemotherapy, *n* (%)	
Yes	430 (93.1)
No	32 (6.9)
Site of pain, *n* (%)^†^	
Head	48 (10.4)
Neck	50 (10.8)
Chest	103 (22.3)
Abdomen	90 (19.5)
Upper back	46 (10.0)
Lower back	107 (23.2)
Joints	56 (12.1)
Others	125 (27.1)
Worst pain intensity over the past 24 hours	
Mean (SD)	4.76 (2.47)
Median (min, max)	5.00 (0.00, 10.00)
Sleep disturbance, *n* (%)	
Yes	253 (54.8)
No	209 (45.2)
Quality of life assessed by EQ-5D-3L	
Mobility, *n* (%)	
No problems	193 (41.8)
Problems	269 (58.2)
Self-care, *n* (%)	
No problems	281 (60.8)
Problems	181 (39.2)
Usual activities, *n* (%)	
No problems	158 (34.2)
Problems	304 (65.8)
Pain/discomfort, *n* (%)	
No problems	82 (17.7)
Problems	380 (82.3)
Anxiety/depression, *n* (%)	
No problems	202 (43.7)
Problems	260 (56.3)

SD: standard deviation; ^†^patients may experience more than one site of pain, percentages may not add up to 100%.

**Table 2:** Patient and physician assessment of satisfaction with pain control (*n*=462). Data presented as *n* (%).

	Patient	Physician
Very satisfied	86 (18.6%)	56 (12.1%)
Satisfied	192 (41.6%)	194 (42.0%)
Acceptable	140 (30.3%)	117 (25.3%)
Dissatisfied	37 (8.0%)	88 (19.0%)
Very dissatisfied	7 (1.5%)	7 (1.5%)

**Table 3:** Total daily dose of analgesics prescribed.

	Median dose (mg)	Minimum, Maximum dose (mg)
Opioids		
Fentanyl^†^	0.89	0.29, 4.49
Morphine	30.00	2.00, 300.00
Tramadol	150.00	30.00, 420.00
Nonopioids		
Gabapentin	900.00	100.00, 3600.00
Paracetamol	1300.00	325.00, 4000.00
Pregabalin	150.00	50.00, 750.00

Only analgesics with data across all six countries were included; all analgesics were orally administered unless otherwise stated; ^†^transdermal.

## Supplementary Materials

Supplementary Materials Supplementary Table 1: patients' and physicians' assessment of satisfaction with pain control by country. Supplementary Table 2: physicians' assessment of adequacy of analgesics by country. Supplementary Table 3: concordance of patients' and physicians' assessment of satisfaction with pain control by country. Supplementary Table 4: total daily dose of opioids prescribed by country. Supplementary Table 5: total daily dose of nonopioids prescribed by country.
