# Supplementary material for: Patient and Physician Satisfaction with Analgesic Treatment: Findings from the Analgesic Treatment for Cancer Pain in Southeast Asia (ACE) Study
Source: Pain Res Manag. 2018 Apr 18;2018:2193710. doi: 10.1155/2018/2193710 (PMC5932441; doi:10.1155/2018/2193710)
Supplement: Supplementary Materials — Supplementary Table 1: patients' and physicians' assessment of satisfaction with pain control by country. Supplementary Table 2: physicians' assessment of adequacy of analgesics by country. Supplementary Table 3: concordance of patients' and physicians' assessment of satisfaction with pain control by country. Supplementary Table 4: total daily dose of opioids prescribed by country. Supplementary Table 5: total daily dose of nonopioids prescribed by country. [file 2193710.f1.docx]

# SUPPLEMENTARY TABLES

| **Supplementary Table 1: Patients’ and Physicians’** **Assessment of Satisfaction with Pain Control by Country** | | | | | | | | | |
| --- | --- | --- | --- | --- | --- | --- | --- | --- | --- |
|  | **Data Set: All Eligible Patients** | | | | | | |  |  |
|  | **All (N=462)** | **Indonesia (N=81)** | **Malaysia (N=99)** | **Philippines (N=105)** | **Singapore (N=7)** | **Thailand (N=100)** | **Vietnam (N=70)** |  | **P** |
| **Satisfaction of pain control status, n (%)** |  |  |  |  |  |  |  |  |  |
| **Assessment by Patient** |  |  |  |  |  |  |  |  | **<0.001*** |
| Very satisfied | 86 (18.6%) | 7 (8.6%) | 7 (7.1%) | 35 (33.3%) | 2 (28.6%) | 29 (29.0%) | 6 (8.6%) |  |  |
| Satisfied | 192 (41.6%) | 41 (50.6%) | 41 (41.4%) | 41 (39.0%) | 2 (28.6%) | 37 (37.0%) | 30 (42.9%) |  |  |
| Acceptable | 140 (30.3%) | 27 (33.3%) | 35 (35.4%) | 23 (21.9%) | 3 (42.9%) | 25 (25.0%) | 27 (38.6%) |  |  |
| Dissatisfied | 37 (8.0%) | 6 (7.4%) | 13 (13.1%) | 6 (5.7%) | 0 (0.0%) | 5 (5.0%) | 7 (10.0%) |  |  |
| Very dissatisfied | 7 (1.5%) | 0 (0.0%) | 3 (3.0%) | 0 (0.0%) | 0 (0.0%) | 4 (4.0%) | 0 (0.0%) |  |  |
| **Assessment by Physician** |  |  |  |  |  |  |  |  | **<0.001*** |
| Very satisfied | 56 (12.1%) | 1 (1.2%) | 12 (12.1%) | 23 (21.9%) | 1 (14.3%) | 18 (18.0%) | 1 (1.4%) |  |  |
| Satisfied | 194 (42.0%) | 19 (23.5%) | 41 (41.4%) | 50 (47.6%) | 4 (57.1%) | 44 (44.0%) | 36 (51.4%) |  |  |
| Acceptable | 117 (25.3%) | 29 (35.8%) | 26 (26.3%) | 22 (21.0%) | 2 (28.6%) | 16 (16.0%) | 22 (31.4%) |  |  |
| Dissatisfied | 88 (19.0%) | 32 (39.5%) | 19 (19.2%) | 10 (9.5%) | 0 (0.0%) | 16 (16.0%) | 11 (15.7%) |  |  |
| Very dissatisfied | 7 (1.5%) | 0 (0.0%) | 1 (1.0%) | 0 (0.0%) | 0 (0.0%) | 6 (6.0%) | 0 (0.0%) |  |  |
| %: column percentage presented  *P-value from simulated Fisher’s exact test for any association between patients’ satisfaction of pain control status across countries | | | | | | | | | |

| **Supplementary Table 2:** **Physicians’ Assessment of Adequacy of Analgesics by Country** | | | | | | | | | |
| --- | --- | --- | --- | --- | --- | --- | --- | --- | --- |
|  | **Data Set: All Eligible Patients** | | | | | | |  |  |
|  | **All (N=462)** | **Indonesia (N=81)** | **Malaysia (N=99)** | **Philippines (N=105)** | **Singapore (N=7)** | **Thailand (N=100)** | **Vietnam (N=70)** |  | **P** |
| **Adequacy of analgesics assessed by Physician, n (%)** |  |  |  |  |  |  |  |  | **<0.001^‡^** |
| Adequate | 329 (71.2%) | 39 (48.1%) | 67 (67.7%) | 88 (83.8%) | 6 (85.7%) | 74 (74.0%) | 55 (78.6%) |  |  |
| Not adequate | 133 (28.8%) | 42 (51.9%) | 32 (32.3%) | 17 (16.2%) | 1 (14.3%) | 26 (26.0%) | 15 (21.4%) |  |  |
| %: column percentage presented  ^‡^P-value from Fisher’s exact test for any association between factors across countries | | | | | | | | | |

| **Supplementary Table 3:** **Concordance of Patients’ and Physicians’ Assessment of Satisfaction with Pain Control by Country** | | | |
| --- | --- | --- | --- |
|  | **Data Set: All Eligible Patients** | | |
|  | **Agreement, n (%)** | **Weighted Kappa (95% CI)** | **P-value from McNemar test** |
| Indonesia (N=81) | 25 (30.9) | 0.13 (0.03, 0.24) | 0.018 |
| Malaysia (N=99) | 44 (44.4) | 0.40 (0.28, 0.53) | <0.001 |
| Philippines (N=105) | 68 (64.8) | 0.59 (0.48, 0.71) | <0.001 |
| Singapore (N=7) | 3 (42.9) | 0.30 (-0.24, 0.84) | 0.266 |
| Thailand (N=100) | 35 (35.0) | 0.28 (0.15, 0.41) | <0.001 |
| Vietnam (N=70) | 35 (50.0) | 0.27 (0.10, 0.44) | 0.001 |
| **All (N=462)** | 45.5 | 0.36 (0.30, 0.43) | <0.001 |
| CI: confidence interval | | | |

| **Supplementary Table 4:** **Total Daily Dose of Opioids Prescribed by Country** | | | | | | | |
| --- | --- | --- | --- | --- | --- | --- | --- |
|  | **Data Set: All Eligible Patients** | | | | | | |
|  | **All (N=462)** | **Indonesia (N=81)** | **Malaysia (N=99)** | **Philippines (N=105)** | **Singapore (N=7)** | **Thailand (N=100)** | **Vietnam (N=70)** |
| **Fentanyl** |  |  |  |  |  |  |  |
| **Transdermal (mg)** |  |  |  |  |  |  |  |
| n (%) | 39 (8.4%) | 14 (17.3%) | 3 (3.0%) | 5 (4.8%) | 1 (14.3%) | 15 (15.0%) | 1 (1.4%) |
| Mean (SD) | 1.30 (1.11) | 1.17 (1.16) | 1.90 (1.48) | 1.13 (1.40) | 1.80* | 1.26 (1.01) | 2.40* |
| Median (Min, Max) | 0.89 (0.29, 4.49) | 0.60 (0.30, 4.49) | 1.20 (0.90, 3.60) | 0.60 (0.29, 3.60) | 1.80 (1.80, 1.80) | 0.89 (0.29, 3.60) | 2.40 (2.40, 2.40) |
| **Morphine** |  |  |  |  |  |  |  |
| **Oral (mg)** |  |  |  |  |  |  |  |
| n (%) | 194 (42.0%) | 39 (48.1%) | 38 (38.4%) | 16 (15.2%) | 4 (57.1%) | 67 (67.0%) | 30 (42.9%) |
| Mean (SD) | 51.08 (50.73) | 25.90 (11.69) | 45.32 (48.24) | 45.00 (44.12) | 53.12 (55.58) | 46.90 (42.51) | 103.42 (68.93) |
| Median (Min, Max) | 30.00 (2.00, 300.00) | 20.00 (15.00, 60.00) | 33.00 (2.00, 300.00) | 30.00 (10.00, 180.00) | 31.25 (15.00, 135.00) | 33.00 (4.00, 200.00) | 60.00 (30.00, 277.50) |
| **Tramadol** |  |  |  |  |  |  |  |
| **Oral (mg)** |  |  |  |  |  |  |  |
| n (%) | 189 (40.9%) | 23 (28.4%) | 41 (41.4%) | 56 (53.3%) | 5 (71.4%) | 32 (32.0%) | 32 (45.7%) |
| Mean (SD) | 139.88 (76.88) | 52.72 (28.94) | 115.55 (54.54) | 127.54 (50.97) | 260.00 (108.40) | 162.89 (68.06) | 213.52 (74.10) |
| Median (Min, Max) | 150.00 (30.00, 420.00) | 37.50 (37.50, 150.00) | 150.00 (37.50, 300.00) | 112.50 (30.00, 300.00) | 300.00 (150.00, 400.00) | 150.00 (50.00, 400.00) | 225.00 (75.00, 420.00) |
| %: column percentage presented  *Standard deviation (SD) is not available when there is only one data.  Patients may be prescribed one or more opioids. | | | | | | | |

| **Supplementary Table 5:** **Total Daily Dose of Non-Opioids Prescribed by Country** | | | | | | | |
| --- | --- | --- | --- | --- | --- | --- | --- |
|  | **Data Set: All Eligible Patients** | | | | | | |
|  | **All (N=462)** | **Indonesia (N=81)** | **Malaysia (N=99)** | **Philippines (N=105)** | **Singapore (N=7)** | **Thailand (N=100)** | **Vietnam (N=70)** |
| **Gabapentin** |  |  |  |  |  |  |  |
| **Oral (mg)** |  |  |  |  |  |  |  |
| n (%) | 97 (21.0%) | 9 (11.1%) | 13 (13.1%) | 9 (8.6%) | 5 (71.4%) | 37 (37.0%) | 24 (34.3%) |
| Mean (SD) | 902.06 (602.25) | 533.33 (250.00) | 900.00 (474.34) | 988.89 (541.86) | 1580.00 (649.62) | 727.03 (658.13) | 1137.50 (508.03) |
| Median (Min, Max) | 900.00 (100.00, 3600.00) | 600.00 (300.00, 900.00) | 900.00 (300.00, 1800.00) | 900.00 (300.00, 1800.00) | 1800.00 (700.00, 2400.00) | 600.00 (100.00, 3600.00) | 900.00 (600.00, 1800.00) |
| **Paracetamol** |  |  |  |  |  |  |  |
| **Oral (mg)** |  |  |  |  |  |  |  |
| n (%) | 126 (27.3%) | 20 (24.7%) | 21 (21.2%) | 23 (21.9%) | 5 (71.4%) | 16 (16.0%) | 41 (58.6%) |
| Mean (SD) | 1651.59 (1087.15) | 469.38 (297.26) | 2277.38 (1253.79) | 1153.80 (518.40) | 3200.00 (836.66) | 1242.19 (544.80) | 2157.93 (927.34) |
| Median (Min, Max) | 1300.00 (325.00, 4000.00) | 325.00 (325.00, 1500.00) | 3000.00 (325.00, 4000.00) | 975.00 (325.00, 2000.00) | 3000.00 (2000.00, 4000.00) | 1100.00 (500.00, 2000.00) | 1950.00 (650.00, 4000.00) |
| **Pregabalin** |  |  |  |  |  |  |  |
| **Oral (mg)** |  |  |  |  |  |  |  |
| n (%) | 46 (10.0%) | 1 (1.2%) | 1 (1.0%) | 34 (32.4%) | 3 (42.9%) | 2 (2.0%) | 5 (7.1%) |
| Mean (SD) | 194.57 (151.65) | 75.00* | 150.00* | 163.24 (92.98) | 650.00 (173.21) | 162.50 (88.39) | 180.00 (41.08) |
| Median (Min, Max) | 150.00 (50.00, 750.00) | 75.00 (75.00, 75.00) | 150.00 (150.00, 150.00) | 150.00 (50.00, 450.00) | 750.00 (450.00, 750.00) | 162.50 (100.00, 225.00) | 150.00 (150.00, 225.00) |
| %: column percentage presented  *Standard deviation (SD) is not available when there is only one data point. | | | | | | | |
